# Arrhythmogenic Right Ventricular Dysplasia (ARVD) With Protein Plakophilin-2 Mutation

**DOI:** 10.7759/cureus.24872

**Published:** 2022-05-10

**Authors:** Maria Riasat, Arshan Khan, Vineet Meghrajani, Mrunalini Gaikwad, Rajwinder Gill

**Affiliations:** 1 Internal Medicine, Icahn School of Medicine at Mount Sinai Beth Israel, New York City, USA; 2 Internal Medicine, Ascension St. John Hospital, Detroit, USA; 3 Cardiology, Icahn School of Medicine at Mount Sinai Beth Israel, New York City, USA

**Keywords:** pkp-2 mutation, right ventricular dilation, ventricular dysrhythmia, heart failure, arvd

## Abstract

Arrhythmogenic right ventricular dysplasia (ARVD) is a heart muscle disease that predominantly affects the right ventricle; however, biventricular involvement is increasingly being recognized. Fibrofatty tissue replacement is a central feature of ARVD. The majority of the identified genes, including protein plakophilin-2 (PKP-2), involved in cell-to-cell adhesion, can be seen in most genetic cases. Clinically, affected individuals present with palpitations, syncope, or sudden death due to ventricular arrhythmias, such as ventricular tachycardia (VT) or fibrillation, with symptomatic heart failure usually only in later stages.

In this study, we present a male patient with ARVD who underwent a genetic test that revealed ARVD with PKP-2 mutation after multiple admissions for heart failure and arrhythmias. He ultimately underwent orthotopic heart transplantation (OHT). Early detection is important for further management, risk stratification, and reduced hospitalization in patients with ARVD.

## Introduction

Arrhythmogenic right ventricular dysplasia (ARVD) is a rare cardiomyopathy characterized by symptomatic arrhythmias and predominantly right ventricular dysfunction [1]. It is a multifaceted disease that can be inherited. ARVD can initially present as sudden cardiac death (SCD) in young adults [2]. One study identifies ARVD as the most common cause of SCD in some areas of Italy, which highlights its significance as inherited cardiomyopathy [3].

## Case presentation

A 71-year-old male with a history of hypertension, hyperlipidemia, ST-elevation myocardial infarction (STEMI) s/p percutaneous coronary intervention (PCI), atrial fibrillation/flutter, and ARVD s/p implantable cardioverter-defibrillator (ICD) presented to the emergency department with acute onset of palpitations. He denied shortness of breath, chest pain, syncope, fever, nausea, and vomiting. Physical examination was noticeable for blood pressure of 134/94 mmHg, heart rate of 130 beats/minute, temperature of 35.4°C, respiratory rate of 18 breaths/minute, and oxygen saturation was 100% on room air. Cardiovascular and pulmonary examinations were unremarkable. Initial laboratory results are given in Table 1.

**Table 1:** Initial laboratory workup.

Test	Results	Reference range
White blood count (WBC)	6.9	4.5-11.00 K/uL
Hemoglobin	15.0	13.6-16.3 g/dL
Platelet	218	150-450 K/uL
Sodium	137	135-145 mEq/L
Potassium	4.3	3.5-5.2 mmol/L
Chloride	96	96-108 mmol/L
Phosphorus	3.3	2.4-4.7 mg/dL
Magnesium	2.1	1.5-2.5 mg/dL
Creatinine	1.68	0.5-1.1 mg/dL
Blood urea nitrogen	25	6-23 mg/dL
Brain natriuretic peptide (BNP)	397.6	0.0-100 pg/mL
Troponin	0.042	<0.031 mg/dL
Aspartate aminotransferase	36	1-35 U/L
Alanine aminotransferase	33	1-45 U/L
Alkaline phosphatase	94	38-126 U/L
pH	7.49	7.35-7.45
pCO_2_	36	35-45 mmHg
Bicarbonate	32	21-29 mEq/L
Lactic acid	1.70	0.50-2.00 mmol/L
Drugs of abuse screen		
Amphetamine screen	Negative	<1000 ng/mL
Benzodiazepine screen	Negative	<200 ng/mL
Cannabinoid (THC) screen	Negative	<50 ng/mL
Cocaine screen	Negative	<300 ng/mL
Methadone screen	Negative	<300 ng/mL
Opiate screen	Negative	<300 ng/mL
Phencyclidine screen	Negative	<25 ng/mL
Barbiturate screen	Negative	<200 ng/mL

Electrocardiogram (ECG) was notable for wide complex tachycardia of 130 beats/minute and left bundle branch block (LBBB). He received magnesium sulfate and amiodarone bolus and was started on an amiodarone drip. The patient's ICD device was interrogated, consistent with atrioventricular (AV) dissociation with slow ventricular tachycardia (VT). Electrophysiologists were consulted who were able to break the VT with programmed electrical stimulation pacing. Patient underwent a transthoracic echo (TTE) which revealed global dilation as well as hypokinesis of the right ventricle. The patient subsequently underwent a right heart catheter which revealed the following filling pressures - right atrium pressure of 12 mmHg, right ventricle pressure of 21/12 mmHg, pulmonary artery pressure of 21/10/14 mmHg, pulmonary capillary wedge pressure of 10 mmHg, Fick cardiac index (Cl) of 1.7 L/min/m^2^. It was determined that he was not a good candidate for the left ventricular assist device (LVAD), and he was placed on a heart transplant list. He underwent left heart catheterization and was found to have a low cardiac output of 1.4 L/min/m^2^ with right ventricular failure with Fontan-like physiology.

The patient was diagnosed with ARVD in 2008 (unknown which criteria for diagnosis were used). Upon further history, the patient revealed that he was originally from a small island in Croatia. He had multiple family members with a similar heart condition as well as a history of sudden cardiac death in his paternal uncle. Patient underwent a genetic test and was found to have a protein plakophilin-2 (PKP-2) mutation and was diagnosed with ARVD. He ultimately underwent orthotopic heart transplantation (OHT) and was started on tacrolimus and mycophenolate mofetil. The patient had a complicated course post-OHT with right ventricular dysfunction and acute kidney injury and also required vasopressor support for the short term. However, after a prolonged hospital course and multiple admissions, the patient is now doing well and continues to follow up for regular checkups and examinations.

## Discussion

Arrhythmogenic right ventricular dysplasia/cardiomyopathy (ARVD/C) is a genetically heterogeneous disease that causes myocardial structural abnormality clinically defined by particular electrical, functional, and structural right ventricular abnormalities characterized by fatty infiltration of the myocardium [1]. Hallmark findings are regional, progressing to global right ventricle dilation. Left ventricle lateral and posterior walls can also be involved in most patients. The mean age of presentation is approximately 30 years. Although it is considered inherited cardiomyopathy, some cases are sporadic, meaning they have neither an identifiable mutation nor a family history of the disease [2-5]. Two patterns of inheritance are identified: autosomal dominant (most common) and recessive (associated with cutaneous findings). Most gene mutation involves desmosomes, which were identified under electron microscopy in the right ventricle of 21 ARVD/C probands [6].

**Figure 1:**
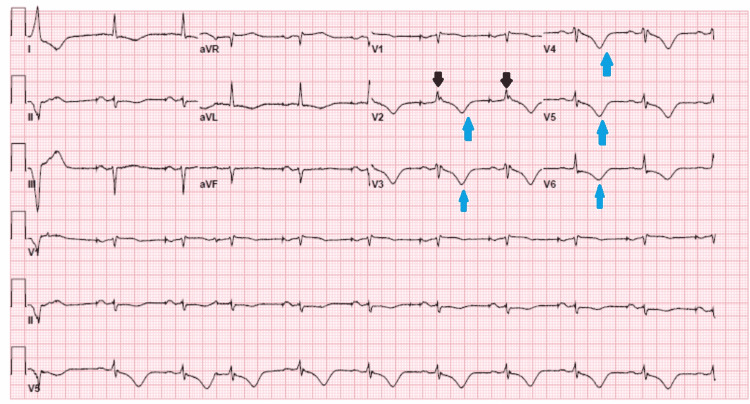
Electrocardiogram demonstrating epsilon wave in lead V2 (evident by black arrows) and T wave inversions evident in V2-V6 (blue arrows).

Differential diagnoses are broad since presenting symptoms are non-specific. However, ARVD should be considered in certain clinical situations, such as patients with unexplained right precordial ECG abnormalities, particularly T wave inversion V1-V3, frequent premature ventricular beats, patients with VT, and LBBB configuration in the absence of apparent heart disease or SCD, especially during exercise [7,8]. Diagnosis can be made using multiple modalities like ECG, transthoracic echocardiogram (TTE), and sometimes cardiovascular magnetic resonance imaging (CMR). The epsilon wave in ECG is a small positive deflection buried at the end of the QRS complex on the ECG is a characteristic of ARVD [7]. Our patient had a similar EKG which can be seen in Figure 1.

Standard guidelines were proposed in 1994 and 2010 by the International Task Force of ARVD to diagnose ARVD/C based on ECG, arrhythmic, morphological, histopathologic, and clinical-genetic factors. The 2010 Task Force criteria require the presence of two major criteria, one major and two minor criteria, or four minor criteria from different categories [9-12]. Management is aimed to prevent SCD with activity restriction, beta-blockers, and ICD. Most patients ultimately require a cardiac transplant. Screening of all first-degree family members is recommended. Prognosis is variable, with the male gender associated with a more malignant course [2].

PKP-2 mutation in arrhythmogenic right ventricular cardiomyopathy

Plakoglobin is a key component of desmosomes and participates in maintaining tight cell-to-cell adhesions. The PKP-2 gene mutation is associated with autosomal dominant ARVD and dilated cardiomyopathy (DCM) [13]. Close relatives (children, siblings, and parents) have up to a 50% chance of being a carrier of this variant. More distant relatives may also be carriers. Parental testing may clarify the inheritance of this variant and may inform recurrence risk. Genetic tests are available; however, these tests should be interpreted with clinical, laboratory, and other imaging modalities.

Our patient’s entire course is summarized as above, he met multiple criteria of ARVD, which includes regional right ventricular dyskinesia as seen in transthoracic echocardiogram (Videos 1, 2).

**Video 1:** ARVD with right ventricle hypokinesia and dilation, four-chamber view (with Definity contrast). ARVD: arrhythmogenic right ventricular dysplasia

**Video 2:** ARVD right ventricle focused view (with Definity contrast). ARVD: arrhythmogenic right ventricular dysplasia

EKG with epsilon waves as mentioned above, a characteristic finding in patients with ARVD, as well as T wave inversions in leads V1-V3 were also evident (Figure 1). The patient belongs to a small island in Croatia where multiple family members had "heart issues." As described above, numerous family members were affected by this condition; however, they never underwent genetic testing like the patient. Interestingly the disease severity varied among family members, some family members had mild symptoms such as palpitations while two of his paternal uncles passed away from SCD. There have not been enough studies conducted on patients who live in Croatia, that would identify the prevalence of SCD in patients who suffer from ARVD. Our patient ultimately had a genetic test and was diagnosed with ARVD with PKP-2 mutation. After a prolonged hospital stay, he eventually underwent orthotopic heart transplantation (OHT).

## Conclusions

With the advancement in heart failure, distinct clinicopathologic entities have been identified with cardiomyopathies. ARVD is one of the inherited cardiomyopathies identified, which can affect multiple family members. PKP-2 is one of the mutations identified with variable penetrance and expressivity. Genetic testing is available and should be offered to patients and family members to manage the disease early.
